# Assessment of jaw muscle activity, bite force and clinical findings in patients with severe tooth wear and matched controls

**DOI:** 10.1038/s41598-026-49563-3

**Published:** 2026-04-21

**Authors:** Maria Erkapers, Susanna Segerström, Peter Svensson, Malin Ernberg

**Affiliations:** 1 Department of Prosthetic Dentistry, Box 1813, Uppsala, Region Uppsala, SE-753 09 Sweden; 2 Department of Dental Medicine, Karolinska Institutet, Box 4064, Huddinge, SE-14104 Sweden; 3 Faculty of Dentistry, National University of Singapore, 9 Lower Kent Ridge Road Level 10, Singapore, 119085 Singapore

**Keywords:** Electromyography, Sleep bruxism, Attrition, Bite force, Saliva flow, Sleep bruxism, Health care, Medical research

## Abstract

The association between tooth wear and sleep bruxism is unclear. This study investigates jaw muscle activity and clinical findings in patients with sleep bruxism and severe tooth wear and matched controls. Participants underwent electromyographic (EMG) recording calibrated for dynamic bite force during sleep and analysed as EMG event duration, events per hour, work volume and accumulated dynamic bite force. In addition, static maximal voluntary bite force, saliva tests, health questionnaires and clinical examination were obtained from all participants. No significant differences were found between groups regarding EMG work volume, accumulated dynamic bite force, or static maximal voluntary bite force. However, the cases had a more EMG events per hour than controls, though controls had longer events than the cases. Groups health data were similar, but the cases had a lower unstimulated saliva secretion. The finding suggest that severe tooth wear and sleep bruxism do not seem to be uniquely related to EMG work volume, accumulated dynamic bite force or static maximal voluntary bite force. However, an increased EMG activity and a marginally decreased unstimulated saliva flow was found and could play a role for severe tooth wear.

## Introduction

Severe tooth wear can cause a variety of individual problems ranging from sensitive teeth and functional problems to loss of teeth. However, aesthetic dissatisfaction and social stigma are often the main reasons for individuals with tooth wear to seek dental advice^1–3^. Among individuals between the age of 18 and 35 years, one of four show clear signs of tooth wear^4^. The relative risk of severe tooth wear increases with age and ranges from 3% at the age of 20 years to 17% at the age of 70 years^5^. Men seems to be more affected by severe tooth wear than women, and has been reported in a ratio of 2.3:1^6^.

There are several underlying mechanisms contributing to tooth wear. Erosion marks the chemical mechanism behind tooth wear while abrasion and attrition describe the physical mechanisms^7,8^. The relative contribution for each factor is not known since available indices do not necessarily measure one specific aetiology, or the study populations may be too diverse in age and clinical characteristics^9,10^. It is also common with a combined effect of attrition and erosion which might potentiate the damage to hard tissue surfaces in the oral environment^9,11,12^.

Attrition is a process where tooth wear is caused by friction when teeth are in contact during function such as chewing, swallowing, and phonetic articulation. The aetiology is multifactorial where several factors have been suggested; increased bite force since men have in general a higher bite force and more attrition than women, a decreased saliva function causing a more erosive and abrasive oral environment, and sleep bruxism (SB) with an increased level of jaw muscle activity^13,14^.

SB is a masticatory muscle activity during sleep that is characterised as rhythmic (phasic) or non-rhythmic (tonic) and is not a movement disorder or a sleep disorder^15^. The evaluation of SB is challenging since it occurs in most people to some extent, and may not be recognized by the individual, making self-reported data unreliable^16^. The fluctuating nature of SB requires an objective measurement to verify increased levels of jaw muscle activity, and this can be obtained by polysomnographic recordings (PSG). It is the most accurate method and has therefore been considered the “gold standard”^17^. However ambulatory electromyographic (EMG) recording devices may also provide sufficient information and often represent a more accessible alternative^18^.

The cause of SB is unknown, but it is likely triggered unconsciously in the brain steam during sleep^19^. The most frequently associated sign to SB is indeed tooth wear^20–22^. However, tooth wear as a unique sign of SB has been questioned due to the multifactorial aetiology of tooth wear and several studies report no significant correlation between tooth wear and level of SB^23–25^.

The diverse results regarding the association between tooth wear and SB is also attributed to the difficulty in diagnosing tooth wear and the limitation of measuring SB objectively. Several attempts have been made to grade tooth wear and identify underlying mechanisms with different indices, such as the Tooth Wear Evaluation Scale (TWES)^12^ which presently seems to be the best instrument for this purpose.

The primary aim of this study was to evaluate the association between jaw muscle activity and force estimates during sleep in patients with possible SB and severe tooth wear. The secondary aim was to evaluate the association between severe tooth wear and additional clinical findings including measures of salivary flow rate. The specific and novel hypothesis was that participants with severe tooth wear have higher accumulated dynamic bite force during sleep than controls with mild or moderate tooth wear.

## Materials and methods

### Ethical aspects

The study followed the guidelines according to the Declaration of Helsinki and was approved by the Regional Ethical Review Board in Uppsala, Sweden 2014-08-27 (2014/307) with amendments 2018-05-16 (2014/307/1) and 2024-06-18 (2024-03750-02). Also, an approval from the local radiation protection committee was obtained 2014-09-01 (D14/13). Written informed consent was obtained from the participants.

### Participants

The study population consisted of cases with severe tooth wear (attrition group) that were referred for oral rehabilitation to the Department of Prosthodontics at the Specialist Clinic Kaniken, Region Uppsala, Sweden, and a control group of gender- and age- (± 2 years) matched volunteers with no, mild, or moderate tooth wear from Uppsala, Sweden.

The inclusion criteria for the cases were age over 18 years, severe tooth wear of at least 1/3 of the dentition primarily caused by attrition according to TWES, a self-reported history of SB audio confirmed by partner, a minimum of 8 teeth/jaw with the possibility of rehabilitating with fixed prostheses at least to a shortened dental arch to ensure similar masticatory function and periodontal sensory feedback before and after dental rehabilitation, and ability to understand and speak Swedish language. The inclusion criteria for the controls were: age over 18 years with no, mild, or moderate tooth wear according to TWES, and complete dentition or absence of single molars that were not planned to be replaced. For both groups the exclusion criteria were alcohol or drug abuse, use of medication with possible effects on sleep or motor behaviour (e.g., benzodiazepine, L-dopa, neuroleptics, and tricyclic antidepressants), neurological or psychiatric disorders, sleep disorders, epilepsy, and less than 8 teeth/jaw.

### Sample size estimation

For sample size calculation, no previous study has been conducted with the bite force prototype, so no pilot data were available. Therefore, the sample size calculation was based on an assumption of an interindividual variability of 25% in the primary outcome measures (EMG activity and dynamic bite force during sleep), a 20% minimal relevant difference, and a risk for type I and II errors of 5% and 20% respectively; this corresponded to 25 individuals per group. To compensate for possible dropouts and missing data (20%), a total of 30 individuals in each group was included.

### General methodology

Cases and controls were first screened. The initial examination included clinical and radiological examination, assessment of tooth wear according to TWES^12^, intraoral photos, and impressions for study casts.

Those who met the requirements after the initial examination received information about the study and were invited to participate. If they accepted, they were asked to complete health questionnaires and subjected to additional examinations including saliva tests, registration of static maximal voluntary bite force (SMVBF) in the awake state, accumulated dynamic bite force (ADBF), and EMG activity during sleep. The clinical examinations were conducted in a clinical environment by an examiner who was trained to handle the SMVBF device by an experienced user.

The ADBF and EMG activity were recorded in the participants home environment, after meticulous instructions.

### Tooth wear evaluation system

The TWES contains several modules including tooth wear grading tools, diagnostic modules, oral history questionnaires regarding oral parafunction, oral dryness, eating habits, reflux, oral hygiene, medications, and treatment modules. This makes it possible to recognize tooth wear, grade its severity and diagnose the underlying cause. The treatment module suggests when to start treatment, what type of treatment that is recommended, and treatment difficulty^12^.

### Saliva test

The saliva samples were collected during various times of the day, at the Department of Prosthodontics at the Specialist Clinic Kaniken according to validated standard methods^26^. In brief, participants were asked not to eat, drink, smoke, or brush their teeth for at least two hours before the test. During the sampling participants were seated with their head inclined forward toward a graded medicine cup for saliva collection and instructed to swallow prior to testing to ensure saliva clearance. Test duration was standardized using a visual timer with audible start and end signals. For stimulated salivary flow, participants chewed standardized wax bilaterally and expectorated continuously during the measurement. For unstimulated salivary flow, participants maintained a slightly open mouth, minimized movement of the tongue and oral musculature to avoid stimulation, and expelled accumulated saliva only at the end of the test. Stimulated saliva was collected for exactly 5 min and unstimulated for 15 min. Saliva volume was quantified using the millilitre graduated collection cup and the flow rate determined by dividing the volume during the time (mL/min).

A stimulated saliva rate of > 0.7 mL/min was considered normal, whereas (< 0.7 mL/min) was defined as hyposalivation. For unstimulated saliva a rate of > 0.25 mL/min was considered normal, whereas (< 0.1 mL/min) was defined as hyposalivation^27^. Buffering capacity and pH were evaluated from stimulated saliva samples by chairside strip test (GC^®^ Saliva Check Buffer^®^, Alsip, USA)^28^. By using a pipette, a drop of the stimulated saliva was placed on a buffer test strip on three test pads. The result was assessed by the colour of the pads after two minutes. Then the result was calculated by adding the points according to the final colour of each pad: green – four points; green/blue – three points, blue – two points, red/blue – one point, red – zero points. All points were counted, and result was determined: 0–5 points as very low buffering ability, 6–9 points as low, 10–12 points as normal/high^28^.

To measure pH, a pH strip was immersed into the unstimulated saliva sample for 10 s. The colour change on the pH strip was then used to estimate the resting pH according to the scale provided by the manufacturer, ranging from red (pH 5.0) to green (pH 7.8).

### Static maximal voluntary bite force

The SMVBF was measured in the awake state with a custom-built U-shaped bite force transducer (Umeå University, Physiology Section, IMB, Umeå, Sweden) with a 11 × 11 mm biting area that was covered with plastic to protect the teeth. The thickness of the device was 6.3 mm (without protective plastic) and 9 mm (with protective plastic). A direct-current whetstone bridge circuit is connected to the bite force transducer and forces from 0 to 1000 N can be recorded^29^. The SMVBF was recorded unilaterally between the molars or premolars depended on the subject’s dentition and ability to bite together. Subjects were instructed to clench their teeth as hard as they could for 3–4 s, and the highest bite force displayed was noted as the peak value. The measurement was done three times with 45 s interval and the average of the three recordings was calculated and used in analyses. Conducting a series of three trials intended to improve reliability and provide a more accurate estimate of the true maximum bite force.

### Accumulated dynamic bite force and electromyographic activity

ADBF and EMG activity were recorded during sleep with a prototype device, GC datalogger (GCDL, Sunstar Suisse SA, Etoy, Switzerland), a modified version of the ambulatory single-channel EMG device BUTLER^®^ GrindCare^®^ (Sunstar Suisse SA, Etoy, Switzerland). The GCDL includes a wireless sensor with three electrode contacts surfaces, a docking station for sensor charging and data transfer, and an accessory software (GCDL PC App) for data interpretation. The sensor was placed on the left anterior temporalis muscle with a double-sided adhesive gel pad. The muscle was identified by the participants by biting and placing their fingers on the protruding muscle. Before use the sensor was calibrated to identify the correlation of EMG and bite force for each participant with a custom-made appurtenant bite force transducer (Sunstar Suisse SA, Etoy, Switzerland) placed in the molar region on the ipsilateral side to the sensor. In cases where severe tooth wear or absence of occluding molars prevented molar placement, the transducer was positioned on the first premolar. Thereafter participants were asked to bite with 2–4 different force levels on the bite force transducer. The different force levels were not defined but corresponded to a light bite, a moderate bite, a strong bite and a very strong bite. To optimize calibration accuracy, participants were carefully instructed about the procedure beforehand and were guided and assisted by the examiner to achieve stable occlusion and with visual feedback of EMG and bite force levels during the biting task.

**Fig. 1:**
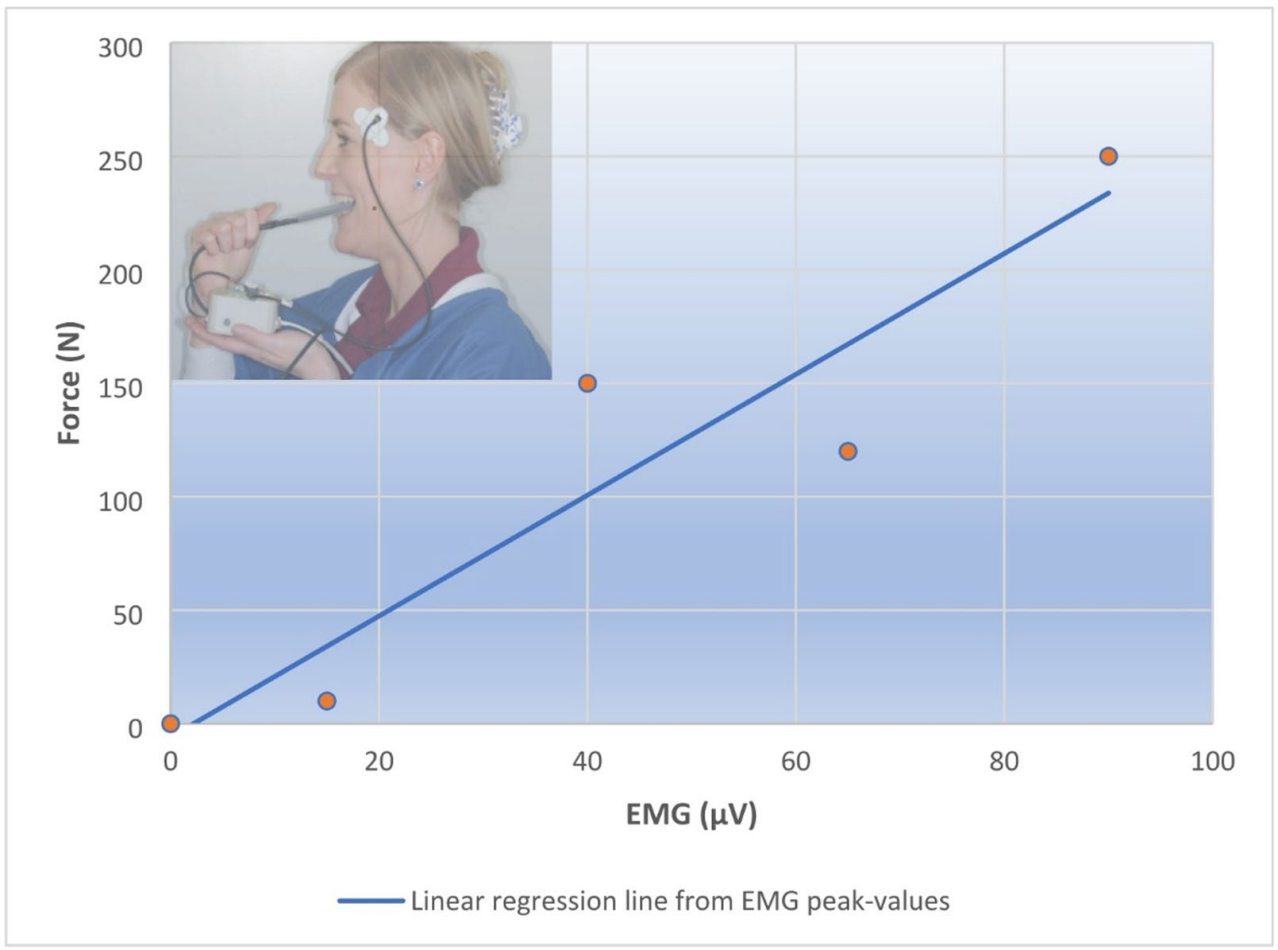
Illustration of GCDL calibration principles regarding EMG and bite force. Participants were asked to bite on a custom-made appurtenant bite force transducer (while connected to the EMG sensor) 2–4 times using a range of bite force for 2–3 s each. An input-output curve was established with EMG peak values and corresponding bite strength values and analysed with linear regression *(Force (N) = EMG (mV . s)* $\:\times\:$ *individual constant (c)).* The generated EMG-bite force curve enabled the individual EMG registration and an estimate of accumulated dynamic bite force and EMG activity during sleep. Consent to publishing identifying information/images in an open access online publication has been obtained.

A correlation between EMG and bite force in this system was determined using the maximum amplitude of the EMG event and of the force signal at each level of the repeated muscle contractions. A linear regression analysis between the 2–4 pairs of EMG and bite force values was done by the GCDL PC App as illustrated in Fig. 1. After calibration, participants were instructed to use the GCDL for 3–5 nights for adequate and representative assessment of jaw muscle activity during sleep^30^. This procedure allowed to obtain an estimate of the force values based on the subsequent recordings of the EMG signal from the temporalis muscle during sleep.

The device analyses EMG activity, according to a signal recognition algorithm, which is based on a fast Fourier transformation analysis and threshold comparison^31,32^. The EMG signal was sampled at 2000 Hz and band-pass filtered (250–610 Hz) prior to signal analysis. The acquisition system used differential amplification with a common-mode rejection ratio (CMRR) of approximately 90 dB. No dedicated notch filter was applied. Power-line interference was minimized through the differential amplifier design and signal acquisition architecture. Electrode-skin contact quality was monitored using an impedance detection algorithm, and recordings were performed only when impedance values were within the acceptable range defined by the recording system (maximum load impedance approximately 5.7 kΩ). Sleep bruxism event detection followed the same general detection framework previously described for BUTLER^®^ GrindCare^®^^30^ including registration of recording time (s), EMG event duration (s) and number of events per hour (n/hour). Workload estimation was based on area integration from the EMG activity and time for registration and calculated above threshold value (mV・s). For ADBF, a conversion of units from mV・s to N was performed using the EMG recordings and a participant-specific calibration constant (c) obtained during the calibration procedure; ($\:EMG\times\:c=N$).

### Statistics

Non-parametric methods were used due to the ordinal character of the data and that Shapiro-Wilk’s tests consistently indicated that variables were not normally distributed. For descriptive statistics, frequency, median, and range where appropriate were used to characterize the sample. For comparison between groups the Fisher´s exact test or Mann-Whitney U tests were used. A P value < 0.5 were considered statistically significant. The processed EMG data were presented as averaged for each of the parameters for three consecutive nights. The data was analysed with SAS version 9.3 on X64.

## Results

### Sample characteristics

Sixty participants were included and completed the study, 30 cases and 30 controls. Each group consisted of 25 men (83.3%) and 5 women (16.7%). Their age range was 25 to 70 years. No statistically significant differences were found between cases and controls regarding demographic data or data from the TWES modules, i.e., type of diet, acid intake frequency, alcohol use, training frequency, reflux problem, or use of medication that can cause oral dryness (Table 1).

**Table 1:** Demographic and background data for 30 cases with severe tooth wear and 30 age- and sex matched controls. Data are presented as numbers (%) or median (range).

Variables	Cases	Controls	*P*
Sex			1
Men	25 (83.3)	25 (83.3)	
Woman	5 (16.7)	5 (16.7)	
Age	59.5 (25–69)	60.5 (28–70)	0.646
Marital status			0.524
Married	25 (83.3)	24 (80.0)	
Divorced	1 (3.3)	3 (10.0)	
Widow/widower	2 (6.7)		
Single		1 (3.3)	
Other	2 (6.7)	2 (6.7)	
Educational level			0.271
High school	4 (13.3)	2 (6.7)	
Gymnasium	14 (46.7)	10 (33.3)	
University	11 (36.7)	18 (60.0)	
Other	1 (3.3)		
Native country			0.849
Sweden	25 (83.3)	27 (90.0)	
The Nordic countries	2 (6.7)	1 (3.3)	
Europe	1 (3.3)		
Other	2 (6.7)	2 (6.7)	
Oral history Questionnaire			
Vegetarian or vegan	3 (10.0)	1 (3.3)	0.612
Acid intake frequency > 2/daily	4 (13.3)	5 (16.7)	0.303
Reflux problem	10 (33.3)	6 (20.0)	0.382
Use of medication which could cause oral dryness	11 (36.7)	5 (16.7)	0.143
Saliva tests			
Unstimulated saliva (ml/min)	0.3 (0–2)	0.4 (0–2)	0.048
Stimulated saliva (ml/min)	1.8 (0–5)	2.0 (1–4)	0.4
Saliva pH*	6.6 (6–8)	6.8 (6–7)	0.326
Buffer capacity pH*	12.0 (5–12)	12.0 (6–12)	0.358

*P* < 0.05 (bold font) denotes significant differences between groups (Mann-Whitney U-test and Fisher’s Exact test) * *n* = 21 cases and 21 controls.

The cases had marginally, but significantly lower unstimulated saliva flow rate compared to controls, but there was no significant difference between groups regarding stimulated saliva flow, buffer capacity, or pH (Table 1).

### Electromyographic activity, work volume, and bite force

**Fig. 2:**
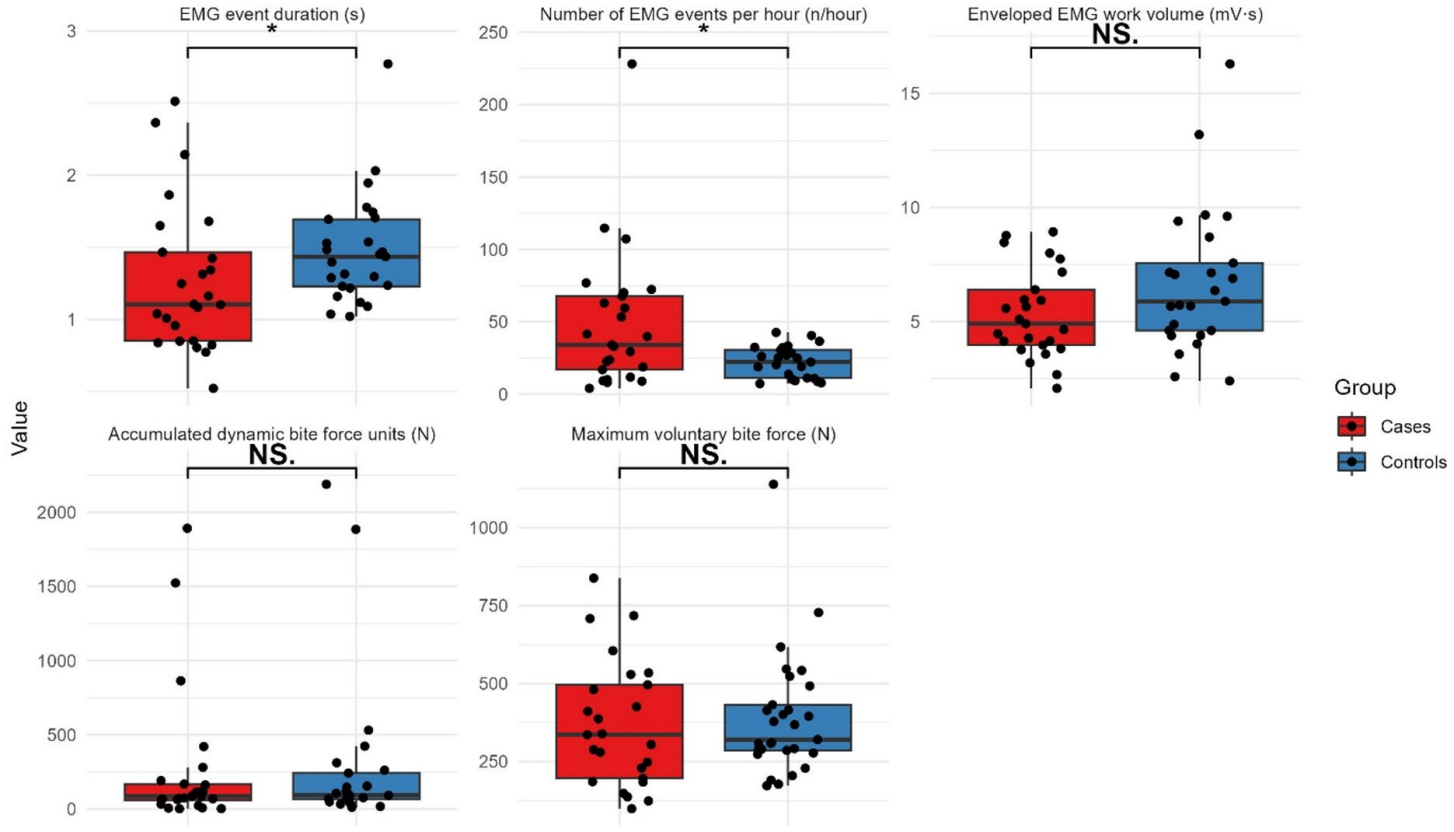
Box plots (median and 95%, 75%, 25%, and 5% percentiles with individual data (dots) illustrating EMG activity and bite forces in cases (*n* = 25) and controls (*n* = 25). * indicate significant difference, NS indicate no significant difference (Mann-Whitney U-test; *P* < 0.05).

The results of the EMG parameters and SMVBF are shown in Fig. 2. No significant differences were found between groups regarding EMG workload or ADBF during sleep, or SMVBF in the awake state. The cases had a higher frequency of EMG events per hour sleep (*P* = 0.035), though EMG event duration was longer for the controls (*P* = 0.029).

The EMG recording length was significantly shorter in cases than in controls (*P* = 0.037), however, no statistically significant between-group differences were observed for the regression constant or SMVBF (Table 2). The relationship between EMG amplitude and bite force obtained during the calibration procedure showed a strong linear association across participants. The median (IQR) coefficient of determination (R²) of the EMG-force regressions was 15,290 (574-11379).

**Table 2:** EMG data for 25 cases with severe tooth wear and 25 age- and sex matched controls. Data are presented as median (range).

Variables	Cases	Controls	*P*
EMG recording			
Registration length (s)	26403.8 (10469–37222)	23864.8 (8008–32428)	**0.037**
Regression constant	15,290 (574–270713)	15,936 (2068–200461)	0.969
**SMVBF** (N) *	304.83 (99–838)	315.67 (172 to 1139)	0.5819

*P* < 0.05 (bold font) denotes significant differences between groups (Mann-Whitney U-test).

* *n* = 30 cases and 30 controls.

## Discussion

The primary aim of this study was to investigate whether severe tooth wear in patients with possible SB was associated with increased jaw muscle activity and ADBF estimates during sleep. No significant difference was found between cases and controls for neither EMG workload or ADBF, and the hypothesis was therefore rejected.

However, the cases had higher frequency of EMG events per hour, though shorter event duration compared to the controls. Both groups displayed a wide range of ADBF values indicating a heterogeneity. The diverse values could be a result of a natural fluctuations in SB at the time of the examination, making the association between tooth wear and the EMG results more complex^25^. The findings from the present study nevertheless indicate that severe attrition cannot solely be explained by higher bite force values during sleep or wakefulness.

Tooth wear as a poor indicator of SB severity in relation to the frequency of jaw muscle activity has previously been confirmed with PSG recordings^33^. This reinforces our findings that tooth wear cannot be simply attributed to masticatory activities during sleep. However, we should be aware that our conclusions emerge from a momentary image of tooth wear which in fact demonstrate a history of accumulated tooth wear. It is likely that tooth wear like device-based sleep bruxism fluctuates over time^15^. This makes evaluation of the two states complex and conclusion should be drawn with caution.

No significant differences were found between groups regarding SMVBF. Our result is in agreement with Cosme et al.^34^. However, our result is in contrast with a previous study^14^. A probable explanation for this is that the previous study included participants with healthy natural dentition while our cases had severe tooth wear and could have avoided to clench with maximum strength due to fear of further damage.

Moreover, no significant differences were found between cases and controls regarding type of diet, acid intake frequency, use of alcohol, training frequency, reflux problem, or use of medication that can cause oral dryness. These diet and health factors are associated with tooth wear due to erosion^35^. The TWES was used to grade tooth wear severity and identify severe attrition in this study, which may partly be explained by successful recruitment.

A secondary finding in this study was that cases had significantly lower unstimulated saliva flow compared to controls. Hypothetically, a lower unstimulated saliva flow would increase tooth friction and attrition during tooth clenching/grinding. A reduced unstimulated saliva flow has also been reported as a risk factor for tooth wear^36^. However, the significance of saliva for tooth wear is unclear^37,38^. Further studies are needed to clarify this. SB has also been suggested as a secondary reflex to physiological variations such as increased or decreased saliva flow^39^ and to stimulate saliva flow as a response to xerostomia to lubricate the oral mucosa. However, its clinical relevance has not been confirmed.

The main strength of this study is that SB was evaluated in a new unique way. The GCDL made calibration between EMG and bite force possible and generated a new way of interpreting SB with work volume and ADBF estimates. This new technique offers a broader concept of analysing SB, not just from number of SB events. Another strength was the use of TWES at inclusion of cases and controls which increases the objectivity and reliability of the study. Also, the exploration of associated factors that can affect tooth wear was extensive and thorough, making the results reliable.

Some limitations, however, need to be acknowledged. The relationship between EMG activity and bite force was established using a bite force transducer combined with linear regression analysis, based on evidence that EMG activity and bite force exhibit a linear relationship^40,41^. However, the proportionality constant between jaw muscle EMG signals and bite force may vary depending on factors such as the presence of temporomandibular disorders and the positioning of the bite force transducer on the occlusal surface^40,42^. To optimize calibration accuracy, participants were instructed and assisted to bite perpendicularly on the transducer in the molar region during calibration. In cases where severe tooth wear or absence of occluding molars prevented molar placement, the transducer was positioned on the first premolar, and participants were guided to achieve stable occlusion. During the calibration process participants had visual feedback (EMG and bite force) during the biting task. However, the use of feedback during the bite-force calibrations might have influenced muscle activation patterns in a manner that does not reflect natural biting behaviour, representing a potential methodological limitation. No feedback was given during SMVBF.

Another methodological limitation is the lack of instrumental and clinical validation regarding correlation between EMG and bite force and ADBF since they were recorded during sleep with a prototype device. However, the prototype is a modified version of the validated ambulatory single-channel EMG device BUTLER^®^ GrindCare^®^. The prototype has been tested in pilot and feasibility settings to verify the stability of EMG recordings, signal processing, and calibration procedures. In the present study, ADBF should therefore be interpreted as an exploratory estimate of cumulative jaw muscle workload derived from calibrated EMG activity rather than as a clinically validated force measurement.

Although this introduced variability in calibration and represents a methodological limitation, it reflects common clinical conditions encountered in the study population.

A further limitation is that potential erosive dietary factors, gastroesophageal reflux, and the use of medications affecting salivary function were evaluated based on self-reported information, which may have introduced reporting bias and resulted in underestimation of their true impact on erosive tooth wear.

The initial saliva tests from nine participants in each group were prepared in mediums and sent to a lab for pH and buffering capacity analyses. The Covid pandemic impeded the lab to proceed this service, and a chairside testing strip test was used instead. The results from the different analyses methods were not comparable, which is the reason why only data from the twenty-one cases and twenty-one controls with chairside testing strip test was used in the final analyses. Also, EMG analyses were made from a subgroup (twenty-five cases and twenty-five matched controls) since five cases, and their matched controls were excluded due to incorrect handling of the bite force transducer causing faulty calibration. This reduced number of participants might have lowered the power. Another limitation in this study is that several outcomes were measured, risking false positive type 1 errors. Avoidance of a type 1 error is desirable in confirmatory studies, however due to the exploratory nature of this study, we did not wish to miss a potentially significant outcome, which is the reason why no Bonferroni correction was done^43^.

Finally, future studies may consider using the Standardized tool for assessment of bruxism (STAB) to comprehensively collect bruxism data^44^. This tool evaluates bruxism status, comorbid conditions, aetiology, and possible consequences. However, it was not available 2019 and could therefore not be used in the present study.

## Conclusion

This study suggests that individuals with severe tooth wear and probable SB do not have an increased ADBF or EMG work volume during sleep, or SMVBF in the awake state compared to controls. However, cases with severe attrition had a higher frequency of EMG events per hour, though a shorter event duration compared to controls. There was a large individual variation in EMG activity among the cases, suggesting a heterogeneity in jaw muscle activity due to natural variance in the SB course. Apart from a marginally lower unstimulated saliva flow in cases with attrition, there were no difference between groups regarding demographic data, diet, or health factors. Severe tooth wear seems to be a multifactorial problem with no single unique risk factor.

## Data Availability

The data that support the findings of this study are not openly available due to reasons of sensitivity and are available from the corresponding author upon reasonable request. Data are located in controlled access data storage at Karolinska Institutet.
